# Navigating the shadows: SlCPK10 mediated flower abscission in tomatoes under low light

**DOI:** 10.1093/plphys/kiae439

**Published:** 2024-09-10

**Authors:** Prateek Jain

**Affiliations:** Plant Physiology, American Society of Plant Biologists; Elysia Bio, 840 Oval Dr, Raleigh, NC 27606, USA

Fruit release requires more than just gravity and involves a complex developmental phase known as “abscission.” The term “abscission” originates from the Latin words “ab” (away) and “sciendere” (to cut), ensuring the shedding of unwanted parts such as post-pollination flowers or senescent leaves. Abscission occurs in the abscission zone (AZ), characterized by cell bundles with dense cytoplasm interconnected by plasmodesmata (Gulfishan et al. 2019). External factors, hormones, and intracellular signaling activate the AZ, crucial for seed and fruit dispersal.

One significant external factor influencing abscission is light (Li et al. 2021). With climate change, understanding the impact of light-related factors—such as duration, intensity, source, and quality—on abscission is crucial for food security. Sunlight fluctuations, cloud cover, leaf movement, and wind reduce light availability, inducing low-light stress. Poor light conditions cause abscission in both vegetative and reproductive organs, ultimately reducing agricultural productivity (Li et al. 2021). Effective management of shade-induced abscission is therefore essential to ensure food security.

In this issue of *Plant Physiology*, Fu et al. (2024) elucidate the role of Inflorescence Deficient in Abscission (IDA/IDLs) and underlying signaling pathways during low-light–induced flower abscission in tomato plants. This study builds on recently published work linking IDA to intracellular calcium levels in *Arabidopsis* (Lalun et al. 2024). The direct involvement of IDA/IDL peptides in abscission make them potential targets for enhancing yield under low-light conditions (Li et al. 2021). To understand the direct role of IDLs in calcium signaling, the authors developed transgenic tomato lines expressing aequorin, a photoprotein and biological calcium indicator (Inouye et al. 1985). Upon treatment with the synthetic SlIDL6 peptide (KPVSGPSKRTNN) at 5 *µ*M, higher calcium levels were observed in the AZ region of transgenic tomato plants, confirming the direct role of IDLs in calcium signaling and AZ activation.

**Figure 1.**
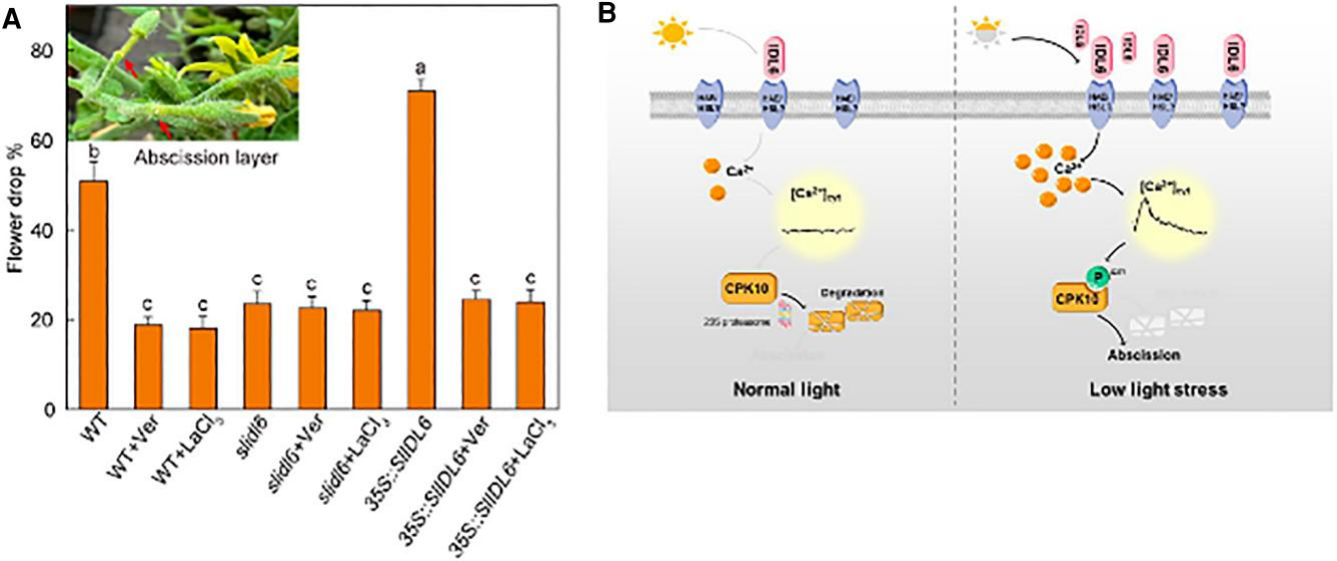
**A)** Plants overexpressing SlIDL6 have higher flower abscission that is reversed after the treatment with Ver and stau. **B)** A working model of SlIDL6 regulated flower drop under low light.

To establish the direct relationship between abscission and SlIDL6-induced calcium signaling under low light, the authors treated aequorin-expressing tomato plants with LaCl_3_ or verapamil (Ver), inhibitors of calcium channels. Plants treated with Ver showed a significant reduction in flower drop compared with wild type (WT) (Fig. 1). Tomato plants overexpressing SlIDL6 exhibited higher flower abscission, which was reversed by Ver treatment. To investigate calcium signaling under low light, the authors examined calcium-dependent protein kinases (CPKs), which detect calcium flux by phosphorylating key proteins (Ray 2017). Treating plants with the CPK inhibitor staurosporine (stau) reduced pedicel abscission induced by SlIDL6 treatment, even under low-light conditions (Tanramluk et al. 2009). Stau treatment also decreased flower drop in SlILD6-overexpressing tomato plants.

The authors further identified genes responsible for SlIDL6-induced flower drop, noting higher expression of *SlCPK6*, *−10*, and *−17*. CRISPR-edited lines of selected *CPKs* confirmed that *slcpk10* has delayed pedicel abscission compared with other genes, with reduced flower drop under low-light conditions. In situ hybridization confirmed abundant *SlCPK10* transcript in AZ, absent under low light in *slidl6* plants. Overexpressing *SlCPK10* lines validated its direct role in promoting flower abscission under both normal and low-light conditions. SlIDL6 treatment had minimal effect on flower drop in *slcpk10* mutants compared with WT, which showed 100% abscission at 32 h, indicating SlCPK10 acts downstream of SLIDL6 during pedicel abscission.

The significance of SlDIL6-regulated calcium signaling under low light prompted the authors to examine the effect on SlCPK10-GFP. They found SlIDL6-induced Ca^2+^ is responsible for post-translational modifications of SlCPK6, which is reversed by Ver treatment. Liquid chromatography-mass spectrometry identified phosphorylation of Ser-371 and Thr-489 motifs in SlCPK10 after SlIDL6 treatment. Transforming *slcpl10* plants with WT SlCPK10 or phospho-dead Ser-371 version confirmed that Ser phosphorylation is crucial for SlCPK10 activation and flower drop under low light.

This study elucidates the critical role of calcium signaling in low-light–induced abscission in tomatoes, demonstrating that SlIDL6 regulates flower abscission through SlCPK10 phosphorylation (Fig. 1). The authors also showed that the application of Ver and Stau suppresses the flower drop triggered by SlIDL6 under low light. In conclusion, the understanding of low-light–induced abscission will help to lower the flower drop and improve food production.
